# Non‐invasive prediction of overall survival time for glioblastoma multiforme patients based on multimodal MRI radiomics

**DOI:** 10.1002/ima.22869

**Published:** 2023-03-10

**Authors:** Jingyu Zhu, Jianming Ye, Leshui Dong, Xiaofei Ma, Na Tang, Peng Xu, Wei Jin, Ruipeng Li, Guang Yang, Xiaobo Lai

**Affiliations:** ^1^ Department of Urology Hangzhou TCM Hospital Affiliated to Zhejiang Chinese Medical University Hangzhou China; ^2^ First Affiliated Hospital Gannan Medical University Ganzhou China; ^3^ School of Medical Technology and Information Engineering Zhejiang Chinese Medical University Hangzhou China; ^4^ The Third Affiliated Hospital Zhejiang Chinese Medical University Hangzhou China; ^5^ Department of Urology Hangzhou Third People's Hospital Hangzhou China; ^6^ Cardiovascular Research Centre Royal Brompton Hospital London UK; ^7^ National Heart and Lung Institute Imperial College London London UK

**Keywords:** deep learning, glioblastoma multiforme, magnetic resonance imaging, overall survival time, radiomics

## Abstract

Glioblastoma multiforme (GBM) is the most common and deadly primary malignant brain tumor. As GBM tumor is aggressive and shows high biological heterogeneity, the overall survival (OS) time is extremely low even with the most aggressive treatment. If the OS time can be predicted before surgery, developing personalized treatment plans for GBM patients will be beneficial. Magnetic resonance imaging (MRI) is a commonly used diagnostic tool for brain tumors with high‐resolution and sound imaging effects. However, in clinical practice, doctors mainly rely on manually segmenting the tumor regions in MRI and predicting the OS time of GBM patients, which is time‐consuming, subjective and repetitive, limiting the effectiveness of clinical diagnosis and treatment. Therefore, it is crucial to segment the brain tumor regions in MRI, and an accurate pre‐operative prediction of OS time for personalized treatment is highly desired. In this study, we present a multimodal MRI radiomics‐based automatic framework for non‐invasive prediction of the OS time for GBM patients. A modified 3D‐UNet model is built to segment tumor subregions in MRI of GBM patients; then, the radiomic features in the tumor subregions are extracted and combined with the clinical features input into the Support Vector Regression (SVR) model to predict the OS time. In the experiments, the BraTS2020, BraTS2019 and BraTS2018 datasets are used to evaluate our framework. Our model achieves competitive OS time prediction accuracy compared to most typical approaches.

## 1 INTRODUCTION

Glioblastoma multiforme, or GBM, is a highly aggressive and deadly type of brain tumor. In 2018, it was estimated that there were approximately 12 760 cases of GBM diagnosed in the United States.^1^ The survival rate for individuals with this type of tumor is bleak, with a median survival time of only 12–15 months. As a result, it is estimated that there are approximately 13 000 deaths due to GBM in the United States each year.^2^ The standard approach to treating GBM typically involves surgery to remove as much of the tumor as possible, followed by radiation therapy and additional chemotherapy. However, due to the high degree of variability in the morphological and genetic makeup of GBM tumors, the response to treatment can be highly varied and the prognosis can vary considerably.^3^ For this reason, early detection of the tumor is crucial in order to improve the chances of a favorable outcome.

At present, the detection and diagnosis of GBM mainly rely on multimodal magnetic resonance imaging (MRI) techniques, which typically have four sequences: T1‐weighted (T1), T1‐weighted contrast enhancement (T1ce), T2‐weighted (T2), and fluid attenuation inversion recovery (FLAIR). Research has shown that brain tumor regions are closely tied to overall survival (OS) time, which requires manual segmentation of the tumor by radiologists.^4^, ^5^ However, manual segmentation is often time‐consuming, subjective, and lacks repeatability, hindering the efficiency of clinical diagnosis. With the advancement of artificial intelligence in medical image analysis, these challenges are being addressed.^6^, ^7^, ^8^, ^9^ The use of medical image analysis techniques allows for quantifying tumor regions and accurately predicting the OS time of GBM patients, providing valuable guidance for personalized diagnoses and treatment plans.

The prediction of the OS time of GBM patients using multimodal MRI images has garnered significant attention from researchers.^10^ Most OS time prediction methods are based on radiomics, which involves analyzing medical image information for disease characterization, tumor grading, and staging. However, to predict the OS time accurately, other factors such as tumor grade must also be taken into consideration. Hence, we aim to achieve OS time prediction for GBM patients through joint representations. This study makes the following contributions: (1) A modified 3D‐UNet network is developed for automatic tumor region segmentation. (2) Both the entire tumor region and three subregions are segmented. (3) The prediction of OS time for GBM patients through joint representations is achieved with superior results.

## 2 RELATED WORKS

### 2.1 Tumor region segmentation

In recent times, the utilization of machine learning algorithms has been prevalent in the field of medical image analysis. Scientists and researchers have been exploring new techniques to detect and segment brain tumors. One such approach is the use of Convolutional Neural Network (CNN) as demonstrated by Altameem et al.^11^ Another study conducted by Xue et al.^12^ employed a cascaded 3D Fully Convolutional Network (FCN) for detecting and segmenting brain metastasis. Ronneberger et al.^13^ introduced U‐Net, a FCN that has been effectively used in biomedical image segmentation. Comelli et al.^14^ proposed using ENet and ERFNet for segmenting aortic aneurysms. Guan et al.^15^ proposed a method for automatic segmentation of brain tumor MR images using the VNet network along with squeezing and excitation modules. They also incorporated an attention guidance filter to mitigate the impact of irrelevant information. Fang et al.^16^ used an improved version of VNet to achieve automatic segmentation of GBM multimodal MRI images, which has significantly enhanced the accuracy and efficiency of clinical diagnosis and treatment.

### 2.2 OS time prediction

In recent years, a significant amount of research has been devoted to the prediction of survival time for cancer patients, particularly with regards to their OS time. A number of studies have employed various methods to achieve this goal. Sun et al.^17^ utilized a 3D‐CNN structure to segment the tumor region, extract image features, and then predict the patient's survival rate. Shboul et al.^18^ took a different approach, extracting texture, volume, and tumor region features and then using recursive feature selection to determine which features were most important. They ultimately employed the XGBoost model to make the prediction of patient survival time. Another study^19^ utilized a generalized linear model to build a predictive model for predicting the prognosis of GBM patients. In this study, the authors incorporated the patient's age into a linear regression model and used the volume feature of the tumor region to make the prediction. Huang et al.^20^ utilized a novel composite method to predict the survival of GBM patients. They acquired a large number of radiomics features, which were then fed into a random forest regression algorithm. Zhou et al.^21^ used quantitative spatial image biomarkers to predict the survival time of GBM patients.

In summary, researchers have employed a range of methods and models to predict the survival of cancer patients, with a focus on the OS time of patients with various types of cancer, including GBM. These methods have included the use of 3D‐CNN structures, image features, texture, volume, tumor region features, recursive feature selection, XGBoost models, linear regression models, composite methods, radiomics features, and quantitative spatial image biomarkers.

### 2.3 Our work

Despite the substantial progress that has been made in the area of automatic segmentation of brain tumors and the prediction of OS time, several obstacles remain that prevent its widespread adoption in clinical settings. The first challenge is the considerable variability in the form, structure, and position of tumors, which makes it challenging to apply a one‐size‐fits‐all approach. Secondly, the data used for analysis often contains significant imbalances between the tumor, the surrounding tissue, and different tumor subregions, making it challenging for automated methods to produce accurate segmentation. Finally, there is a lack of integration of important factors such as tumor grade and patient age into radiomics‐based models, which could further improve the predictive accuracy of these models.

Our work aims to tackle the aforementioned challenges by introducing a novel framework for the non‐invasive prediction of OS time in patients with GBM using multimodal MRI radiomics. The framework consists of two key steps. Firstly, it employs a modified version of the 3D‐UNet model for segmenting three subregions in multimodal MRI scans. This is followed by the extraction of radiomic features from the segmented images. In the second step, these features are combined with relevant clinical information to create a predictive model based on SVR. The final result is an OS time prediction for GBM patients, which can be achieved without the need for invasive procedures.

## 3 METHODS

### 3.1 Data preprocessing

We utilize the Z‐score approach to normalize the image, which involves processing the data by subtracting the mean value of the image's pixel values and dividing by the standard deviation. The Z‐score calculation formula is as follows (1):

(1) $$Z=\frac{X-\bar{X}}{s}$$

where Z indicates the image matrix after normalization, X indicates the original image matrix, $\bar{X}$ denotes the mean of the pixel, and *s* denotes the standard deviation of the image.

**FIGURE 1:**
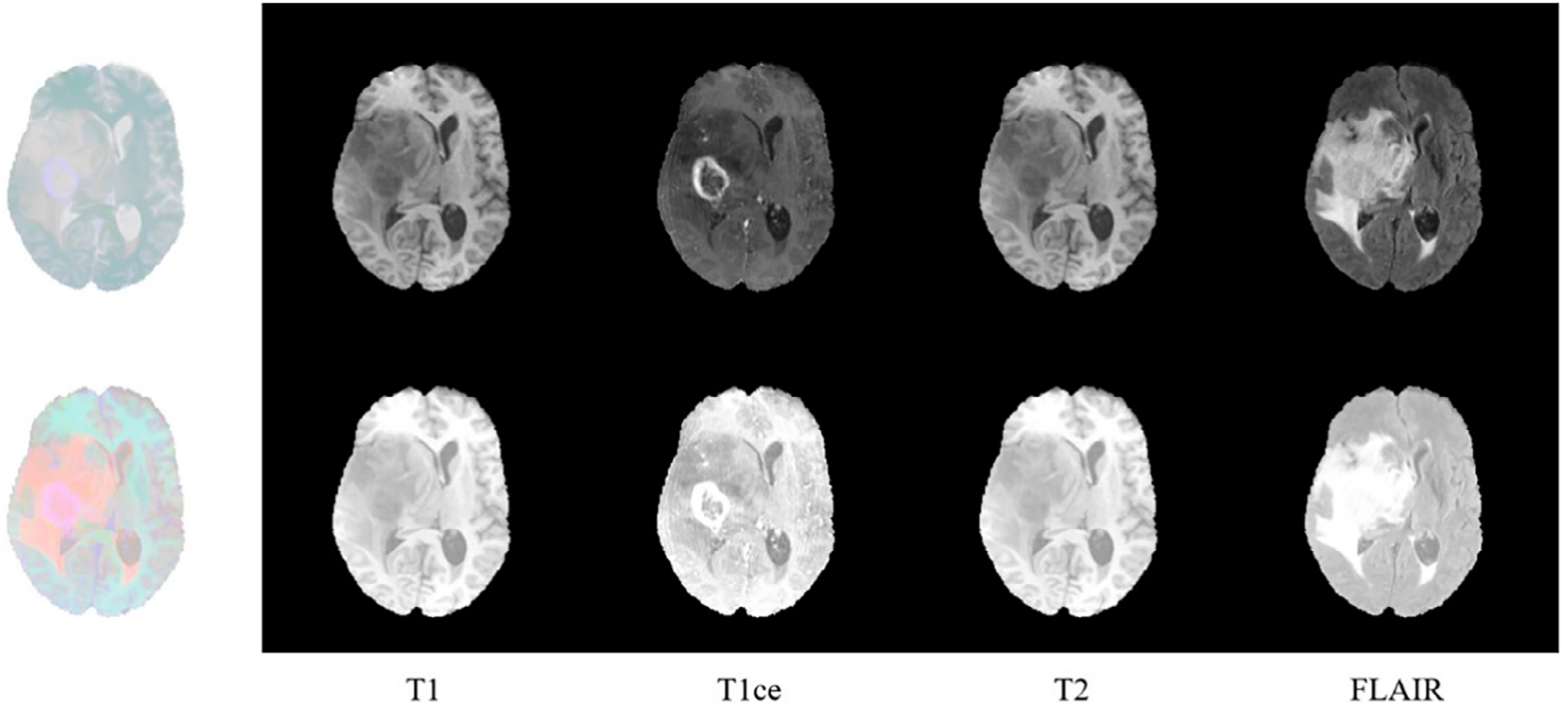
Comparison of results before and after preprocessing.

The impact of the preprocessing procedure is demonstrated in Figure 1. The comparison of the four sequences, T1, T1ce, T2, and FLAIR, both before and after preprocessing, is displayed in columns 2–5. The combined effect of the preprocessing on all four sequences is shown in the first column. As seen in Figure 1, the contrast of the tumor region is improved after preprocessing, making it easier to segment the GBM subregions.

We improve edge detail detection by cropping the individual samples after preprocessing. The original size of 240 × 240 × 155 is reduced to 128 × 128 × 128, keeping only the samples with labeling in the dataset and discarding images containing only unlabeled lesion regions. This step is necessary because the majority of images in the brain tumor datasets used in this study consist of background regions, with only a small portion depicting the tumor regions. It poses a challenge for accurate brain tumor segmentation as the model could be biased towards the background, which is the majority class, and not perform well on the minority class, the tumor regions.

### 3.2 Segmentation framework

**FIGURE 2:**
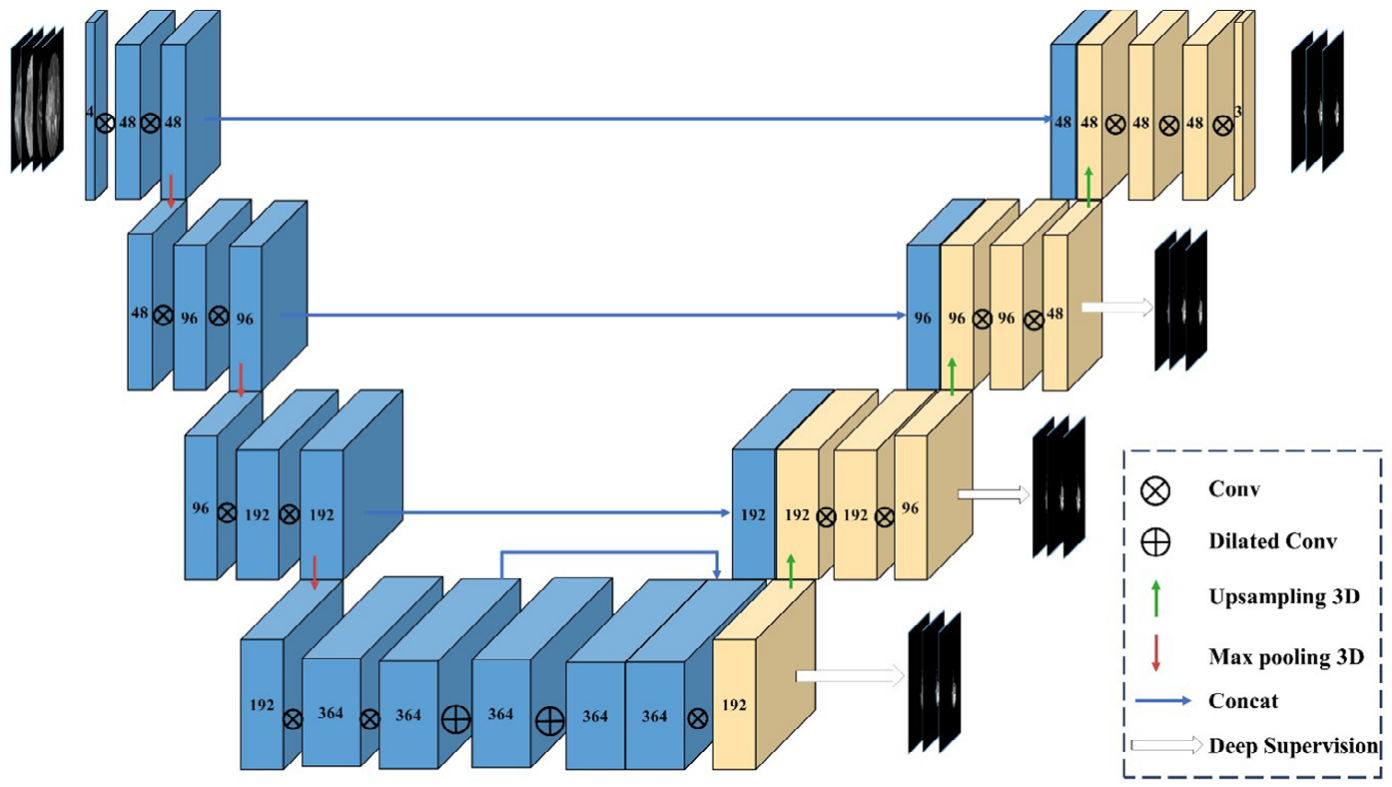
Schematic diagram of modified 3D‐UNet model structure.

In this study, a modified version of the 3D‐UNet architecture was developed to segment three subregions of GBM in multimodal MRI scan data. The architecture consists of three distinct components: an encoder, a decoder, and a concatenation, as depicted in the overall structure diagram in Figure 2. These components are described in greater detail in the subsequent sections of the study.

#### 3.2.1 Encoder

A modified 3D‐UNet model was created for the segmentation of three GBM subregions in multimodal MRI scans. The model consists of three main components: encoder, decoder, and concatenation. The structure of the model is depicted in Figure 2 and each component is described in further detail. The encoder comprises of four stages, with each stage consisting of two 3 × 3 × 3 convolutions followed by a normalization layer and a nonlinear activation layer using ReLU. Instead of Batch Normalization, the model uses Group Normalization and Instance Normalization. The first convolution in each stage increases the number of filters while the second convolution maintains the output's channel count. A MaxPool layer is added between each stage, downsampling the space and increasing the number of filters by one after each pooling. The kernel size in the MaxPool layer is 2 × 2 × 2 with a stride of 2. After the final stage, two 3 × 3 × 3 inflated convolutions with an expansion rate of 2 are applied, and the output of the last stage is concatenated.

#### 3.2.2 Decoder

The decoder is designed to complement the encoder with a similar structure and uses trilinear interpolation to resize the feature maps between each stage. The encoder and decoder are concatenated at the same spatial resolution. The final layer of the decoder consists of three output channels, a sigmoid activation, and a 1 × 1 × 1 kernel size. The lowest spatial resolution is achieved using a 3 × 3 × 3 convolution in the last stage of the encoder.

#### 3.2.3 Loss function

In medical image segmentation, the Dice similarity coefficient (DSC) is often used to measure the degree of overlap between the ground truth and the predicted image. The expression for calculating the Dice similarity coefficient is shown in Equation 2:

(2) $$\mathrm{DSC}=\frac{2\left|X\cap Y\right|}{\left|X\right|+\left|Y\right|}$$

where *X* denotes the predicted value, and *Y* denotes the true value.

The entire tumor, tumor core, and enhanced tumor regions are optimized with Dice loss, after which the Dice loss function for each region is summed to yield the final loss. The soft dice loss expression is given in Equation 3:

(3) $$\text{Loss}=\frac{2\sum \mathrm{XY}}{\sum X^{2}+\sum Y^{2}+\varepsilon }$$

where ε is the smoothing factor (in our experiments this factor is set to 1).

### 3.3 OS time prediction

**FIGURE 3:**
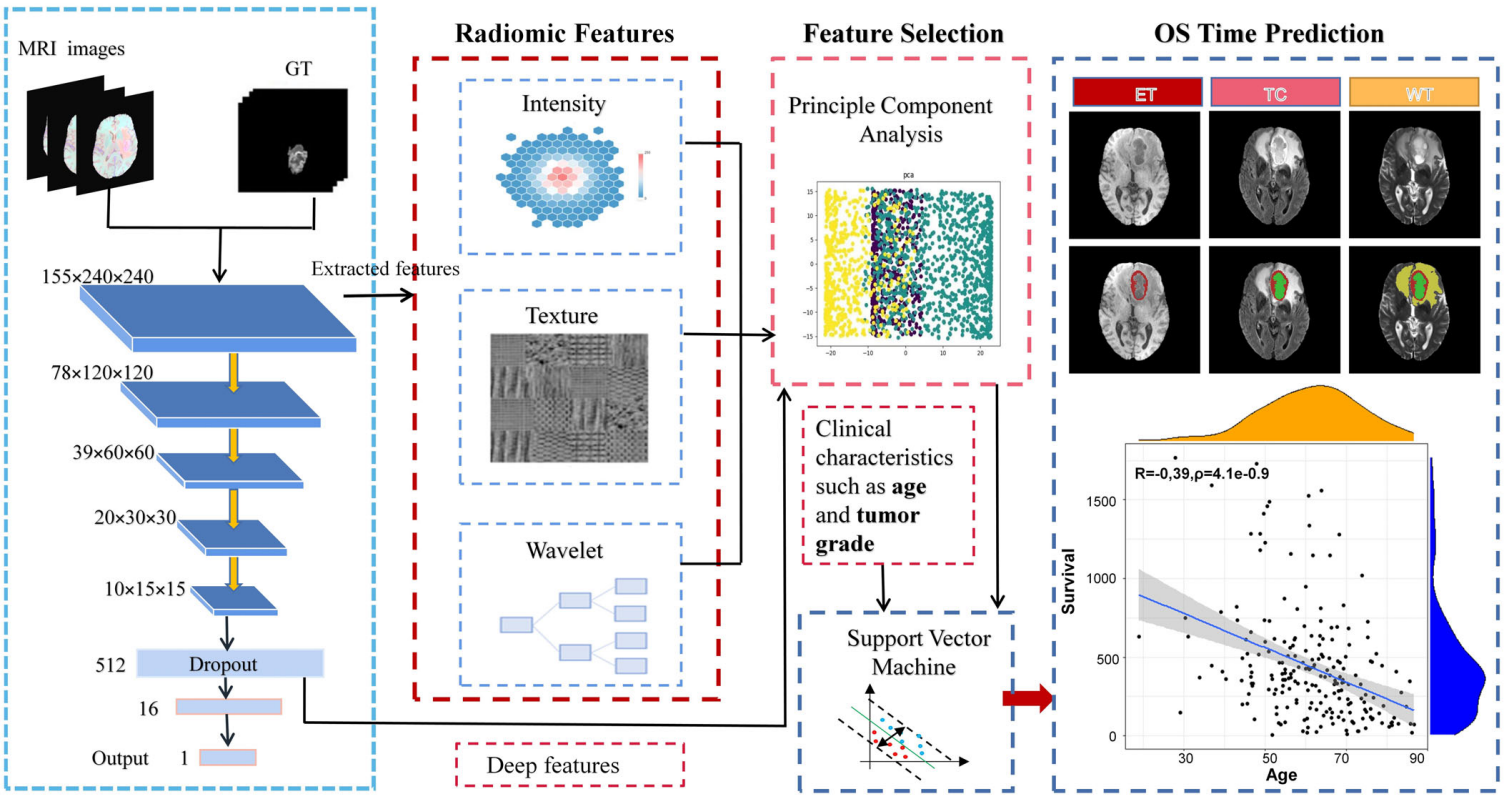
Flow chart of the survival prediction model.

We present a framework for non‐invasive OS time prediction of GBM patients using multimodal MRI radiomics. The process is shown in a flow chart in Figure 3. Radiomic features including intensity, texture, and wavelet are extracted, followed by a CNN to extract deep features. Both types of features are selected using Principal Component Analysis (PCA) to remove redundant information. Finally, the selected features and clinical parameters like age and tumor grade are combined and used as input to the SVR model to predict the OS time.

#### 3.3.1 Feature extraction

We utilize the PyRadiomics toolbox to extract radiomic features from the segmented subregions of the GBM tumor. These features are based on the results of the segmentation process and include intensity, texture, and wavelet features, which are further classified into seven categories. The Gray‐Level Co‐occurrence Matrix (GLCM) is used to analyze the texture of the tumor by analyzing the spatial correlation between gray levels and providing information on various aspects such as correlation, energy, contrast, defect, variance, probability, entropy, and the sum of squares. The first‐order statistical features describe the distribution of voxel intensities within the image region defined by the mask. Shape features, including sphericity, perimeter ratio, spindle length, and elongation, are also calculated to describe the 3‐dimensional form of the tumors. Additionally, 14 Gray Level Dependency Matrix (GLDM) features, 5 interpolated statistic features, 16 features from the Gray Level Size Zone Matrix (GLSZM), and 16 from the Gray Level Run Length Matrix (GLRLM) are extracted. A comprehensive list of the features included in each category can be found in Table 1.

**TABLE 1:** Categories and specific content of features extracted.

Feature category	Feature name
Shape (13)	Elongation, Flatness, Least Axis Length, Major Axis Length, Maximum 2D Diameter (Column), Maximum 2D Diameter (Row), Mesh Volume, Minor Axis Length, Sphericity, Surface Area, Surface Volume Ratio, Voxel Volume Maximum 2D Diameter (Slice), Maximum 3D Diameter.
First order (18)	10Percentile, 90Percentile, Mean, Median, Energy, Entropy, Interquartile Range, Total Energy, Uniformity, Kurtosis, Maximum, Mean Absolute Deviation, Minimum, Range, Robust Mean Absolute Deviation, Root Mean Squared, Skewness, Variance.
GLCM (22)	Autocorrelation, Inverse Variance, Cluster Shade, Correlation, Difference Average, Sum Entropy, Joint Energy, Cluster Tendency, Contrast, Joint Entropy, Imc1, Imc2, Idm, Idmn, Id, Idn, Maximum Probability, Difference Variance, Difference Entropy, Sum Squares, Joint Average, Cluster Prominence.
GLRLM (16)	Gray Level Non‐Uniformity, High Gray Level Run Emphasis, Long Run Emphasis, Long Run Low Gray Level Emphasis, Low Gray Level Run Emphasis, Run Entropy, Run Length Non‐Uniformity, Run Length Non‐Uniformity Normalized, Gray Level Variance, Run Percentage, Long Run High Gray Level Emphasis, Run Variance, Short Run Emphasis, Short Run High Gray Level Emphasis, Gray Level Non‐Uniformity Normalized, Short Run Low Gray Level Emphasis.
GLSZM (16)	Gray Level Non‐Uniformity, High Gray Level Zone Emphasis, Large Area Emphasis, Gray Level Variance, Large Area Low Gray Level Emphasis, Low Gray Level Zone Emphasis, Size Zone Non‐Uniformity, Gray Level Non‐Uniformity Normalized, Size Zone Non‐Uniformity Normalized, Small Area High Gray Level Emphasis, Small Area Low Gray Level Emphasis, Small Area Emphasis, Large Area High Gray Level Emphasis, Zone Entropy, Zone Percentage, Zone Variance.
GLDM (14)	Dependence Entropy, Dependence Variance, Gray Level Non‐Uniformity, High Gray Level Emphasis, Large Dependence High Gray Level Emphasis, Low Gray Level Emphasis, Large Dependence Low Gray Level Emphasis, Small Dependence High Gray Level Emphasis, Small Dependence Emphasis, Gray Level Variance, Dependence Non‐Uniformity, Small Dependence Low Gray Level Emphasis Large Dependence Emphasis Dependence Non‐Uniformity Normalized.
Interpolated statistic (9)	Spacing, Bounding Box, Voxel Num, Volume Num, Center Of Mass Index, Center Of Mass, Mean, Minimum, Maximum.

Moreover, our method involves collecting features from both the original MRI image and a version of the image that has undergone wavelet decomposition. This approach is designed to provide a comprehensive set of characteristics for the tumor subregions. The wavelet decomposition process divides the image into multiple levels of detail, which enables the extraction of a total of 2500 image features. This combination of features from the original image and the wavelet decomposition enhances the accuracy and robustness of the prediction model.

In our newly proposed CNN network, the final fully connected layer is specifically designed to predict the OS time of GBM patients. The network has a structure that comprises four convolutional layers with a stride of 2, and three fully connected layers. The architecture is not only capable of extracting deep information from the MRI images, but also of directly estimating the number of survival days. After training, the network can extract 512 deep features, which can be used for further analysis. The combination of these deep features and the imaging features is then subjected to a feature selection process to eliminate redundant features and improve the accuracy of the prediction model. Additionally, the CNN network has the capability to learn the shape and texture characteristics of brain tumors, which are important imaging properties that can impact the prediction of OS time.

#### 3.3.2 Feature selection

In the process of extracting features, some of the extracted features may be redundant or unimportant for OS time prediction, resulting in overfitting of the model. To address this issue, we use PCA for feature selection to decrease the dimension of the data set and retain the features that have the highest impact on the squared difference of the data set. This helps in analyzing small sample sizes and high‐dimensional, high‐volume data. The main steps involved in the PCA calculation are as follows:

The sample of $x={\left(x_{1},x_{2},\ldots ,x_{p}\right)}^{T}$ dimension is transformed into the standard matrix of *p* dimension, as shown in the following formula:

(4) $$Z_{\mathrm{ij}}=\frac{x_{\mathrm{ij}}-{\bar{x}}_{j}}{s_{j}},1,2,\ldots ,n,j=1,2,\ldots ,p$$

Where *Z* is the standardized matrix.

And the correlation coefficient matrix is solved for Z, the calculation formula is as follows:

(5) $$R={\left[r_{\mathrm{ij}}\right]}_{p}\mathrm{xp}=\frac{Z^{T}Z}{n-1}$$

Where $r_{\mathrm{ij}}=\frac{\sum z_{\mathrm{kj}}\cdot z_{\mathrm{kj}}}{n-1}$, i,j=1,2,…,n.

Then, make the characteristic equation $|R‐\lambda I_{p}|=0$ of the sample correlation matrix *R*, to obtain *p* characteristic roots. The specific value of M is determined by the formula $\frac{\sum_{j=1}^{m}\lambda_{j}}{\sum_{j=1}^{p}\lambda_{j}}\geq t$ to make the information utilization rate reach more than *t*. for each $\lambda_{j}$, the unit eigenvector $b_{j}^{0}$ is obtained by solving $\mathrm{Rb}=\lambda_{j}b$.

Finally, the standardized index variable is converted into the main component, and its calculation method is as follows:

(6) $$U_{\mathrm{ij}}=z_{i}^{T}b_{j}^{0}$$

Where $U_{p}$ 1 is regarded as the *p*th principal component. The variance contribution rate of each principal component is used to weight and sum the m principal components.

#### 3.3.3 Prediction model

After the feature selection, our framework obtained a set of effective features for OS time prediction. To ensure comprehensive feature coverage, clinical features like age and tumor grade are included as essential components of the survival prediction features, which are combined with the effective feature set and fed into the SVR model to predict the OS time of GBM patients. The SVR model, which utilizes SVMs for regression, is widely used in response prediction according to the literature review (cited in^22^, ^23^, ^24^). While the SVR model has a strong generalization ability and fast training speed, there is room for improvement, as evidenced by an improved SVR algorithm for survival analysis proposed by Shivaswamy et al.^25^ This improved algorithm maintains the advantages of the support vector method while enhancing the ordinary model. With the combination of effective features and clinical features, the SVR model can predict the OS times of GBM patients accurately and efficiently.

The SVR model works by mapping the input vector into a high‐dimensional space through nonlinear transformation and constructing the regression function in this space based on the principle of structural risk minimization.^26^ Given *r* data samples ${\left\{x_{i},y_{i}\right\}}_{i=1}^{r}$, where *x* are the input samples and *y* are the output samples, the model maps the inputs from the original space into an *M*‐dimensional feature space to create a hyperplane or approximation function. The calculation is as follows:

(7) $$f\left(x\right)=\sum_{i=1}^{M}\omega_{i}\Phi_{i}\left(x\right)+b$$

Where b is the offset. After Φ‐transform, the input samples can solve the linear regression problem in the high‐dimensional space to achieve the goal of solving the nonlinear regression problem in the original space.

## 4 DATASET AND SETTING

### 4.1 Datasets

Our model is evaluated using the BraTS2020, BraTS2019, and BraTS2018 datasets,^27^, ^28^ which contain two categories of brain tumors: High‐grade glioma (HGG) and low‐grade glioma (LGG), each with different biological characteristics. Each sample in the datasets contains images from four imaging modalities: T1, T1ce, T2, and FLAIR, which have different signal intensities, textures, and spatial information. The subregions of the tumor that are evaluated included the whole tumor (WT), enhanced tumor (ET), and tumor core (TC) regions, with the aim of achieving an automated segmentation of these subregions. The validation sets in the BraTS2020, BraTS2019, and BraTS2018 datasets consist of 125, 125, and 66 cases respectively, while the training sets consist of 369, 335, and 285 samples, respectively.

### 4.2 Evaluation metrics

We evaluate the segmentation results quantitatively using five metrics: the Dice score (Dice), sensitivity, specificity, Hausdorff95 distance (Haus95), and average boundary displacement (ABD). These metrics are used to verify the accuracy of the model's segmentation. Sensitivity measures the number of correctly segmented tumor subregions, while specificity measures the number of correctly segmented normal tissue regions. The Haus95 distance, which eliminates the impact of outliers, is calculated as the 95th percentile of the distances between actual and predicted values. ABD measures the average distance between corresponding points on the boundaries of the ground‐truth (GT) and predicted segmentation masks, with a smaller score indicating a better match. The calculation methods are listed in formulas (8) (12).

(8) $$\text{Dice}=\frac{2\mathrm{TP}}{\mathrm{FP}+2\mathrm{TP}+\mathrm{FN}}$$

(9) $$\text{Sensitivity}=\frac{\mathrm{TP}}{\mathrm{TP}+\mathrm{FN}}$$

(10) $$\text{Specificity}=\frac{\mathrm{TN}}{\mathrm{FP}+\mathrm{TN}}$$

(11) $$\begin{array}{l} \text{Hausdorff}95\left(X,Y\right)\\ \;=\max\left\{d_{\mathrm{XY}},d_{\mathrm{YX}}\right\}\\ \;=\max\left\{\max_{x\in X}\underset{y\in Y}{\min d}\left(x,y\right),\max_{y\in Y}\underset{x\in X}{\min d}\left(x,y\right)\right\} \end{array}$$

(12) $$\mathrm{ABD}\left(X_{s},Y_{s}\right)=\begin{array}{l} \frac{1}{\left|X_{s}\right|+\left|Y_{s}\right|}(\sum_{x_{t}\in X_{s}}\min_{y_{t}\in Y_{s}}\left\|x-y\right\|\\ +\;\sum_{y_{t}\in Y_{s}}\min_{s\in X_{s}}\left\|x-y\right\|) \end{array}$$

Where *TP* is the number of correctly segmented tumor subregions, *FP* is the number of normal tissues incorrectly labeled as tumor subregions, *FN* is the number of tumor subregions incorrectly labeled as normal tissues, and *TN* is the number of normal tissue regions predicted to be tumor subregions. *X*_*s*_ denotes the prediction region surface, *Y*_*s*_ denotes the GT, and ||*x*_*t*_ ‐ *y*_*t*_|| represents the Euclidean distance between voxels *x*_*t*_ and *y*_*t*_.

We evaluate the accuracy of the survival prediction model using three metrics: mean square error (MSE), mean absolute error (MAE), and root mean square error (RMSE). MSE calculates the square of the difference between the predicted and actual values, MAE adds up the absolute differences between the predicted and actual values, and RMSE is the square root of the ratio of the squared deviation between the predicted and actual values and the number of repetitions. These three metrics all measure the difference between the predicted and actual values. The formulas for calculation are displayed in (12–14).

(13) $$\mathrm{MSE}=\frac{1}{n}\sum_{i=1}^{n}{\left(X_{\mathrm{obs},i}-X_{\mathrm{pre},i}\right)}^{2}$$

(14) $$\mathrm{MAE}=\frac{1}{n}\sum_{i=1}^{n}|X_{\mathrm{obs},i}-X_{\mathrm{pre},i}|$$

(15) $$\text{RMSE}=\sqrt{\frac{\sum_{i=1}^{n}{\left(X_{\mathrm{obs},i}-X_{\mathrm{pre},i}\right)}^{2}}{n}}$$

Where *i* denotes for *i*‐th patients, *n* denotes for the total number of patients, $X_{\mathrm{obs}}$ denotes for the real survival time of patients, and $X_{\mathrm{pre}}$ denotes for the survival time predicted by the model.

### 4.3 Experimental details

We first normalize the original image data using Z‐score normalization and then crop the images to a variable size using a bounding box that encompassed the entire brain. The model is trained for a maximum of 400 iterations, and the model with the lowest loss on the validation set is saved as the best model. The model is trained using a batch size of three and the Adam optimizer with an initial learning rate of le‐4.

To ensure the robustness of the model, we employ a cyclic cross‐validation approach to evaluate and test the accuracy and reliability of the model in predicting the OS time of GBM patients. The dataset is divided into a training set and a test set, with the model first trained on the training set and its performance evaluated using the test set. This process is repeated 100 times, with the data being randomly split into training and test sets each time, and with the training set accounting for 0.9 of the total data and the test set accounting for 0.1 of the total data. The final error loss is calculated as the average of the 100 cross‐validations, ensuring that different data combinations are used for training and testing and that the results of each iteration are verified.

The experiments are carried out using Pytorch on a computer with an Intel Xeon Gold 6226R CPU @ 2.90GHz with 16 cores and 4 NVIDIA RTX A5000 GPUs, each with 24GB of memory. The software platform used for development is PyCharm with Python 3.6, and the packages utilized are SimpleITK 1.2.4, NumPy 1.19.1, scikit‐learn 0.23.1, pyyaml 5.3.1, pandas 1.0.3, and scipy 1.4.1.

## 5 RESULTS AND DISCUSSION

### 5.1 Results

#### 5.1.1 Segmentation results

Our model is trained on the training set and then tested on the corresponding validation set of three brain tumor segmentation datasets. The objective of the task is to segment the tumor subregions for evaluation, which include the WT, ET, and TC. Table 2 shows the average results of our model on the BraTS2020 training and validation sets.

**TABLE 2:** Outcomes of metrics for different tumor subregions on the BraTS2020 dataset.

Data Set	Dice			Sensitivity			Specificity			Haus95			ABD		
	ET	WT	TC	ET	WT	TC	ET	WT	TC	ET	WT	TC	ET	WT	TC
Training	0.747	0.881	0.828	0.777	0.943	0.860	0.999	0.999	0.999	30.8	13.7	7.0	7.68	6.40	2.74
Validation	0.691	0.832	0.729	0.767	0.929	0.821	0.999	0.999	0.999	37.1	19.7	16.6	11.2	10.1	6.87

The results shown in Table 2 indicate that the proposed model achieved exceptional performance based on the evaluation indices. A result closer to 1 reflects better segmentation performance. The model demonstrated a specificity index of 0.999, which indicates high accuracy in segmenting normal tissue regions. The accuracy of segmentation for the largest and most prominent region (WT) is higher in the training set compared to the validation set due to the constant refinement of the training data. The low value of Hausff95, which represents the maximum difference between the segmentation result and the GT label, highlights the precise boundary segmentation results achieved by the model.

Our model is also evaluated on the BraTS2019 and BraTS2018 datasets to determine its reliability and efficiency. The results, as shown in Table 3, indicate that the model produced consistent outcomes on both datasets, demonstrating its validity. However, there may be some variations in the performance due to differences in data size, sample information, and other factors. The best performance is observed on the BraTS2018 dataset.

**TABLE 3:** Outcomes of various metrics on the BraTS2019 and BraTS2018 datasets.

Dataset		Dice			Sensitivity			Specificity			Haus95			ABD		
		ET	WT	TC	ET	WT	TC	ET	WT	TC	ET	WT	TC	ET	WT	TC
Training	2019	0.79	0.87	0.86	0.89	0.94	0.91	0.99	0.99	0.99	9.80	16.7	6.15	3.23	7.02	2.29
	2018	0.70	0.83	0.87	0.85	0.93	0.86	0.99	0.99	0.99	9.95	16.6	6.38	2.71	7.41	2.45
Validation	2019	0.75	0.79	0.72	0.71	0.93	0.75	0.99	0.99	0.99	18.7	17.8	20.8	8.82	7.71	9.61
	2018	0.66	0.83	0.75	0.72	0.93	0.76	0.99	0.99	0.99	13.7	20.5	16.5	6.40	8.54	5.63

**FIGURE 4:**
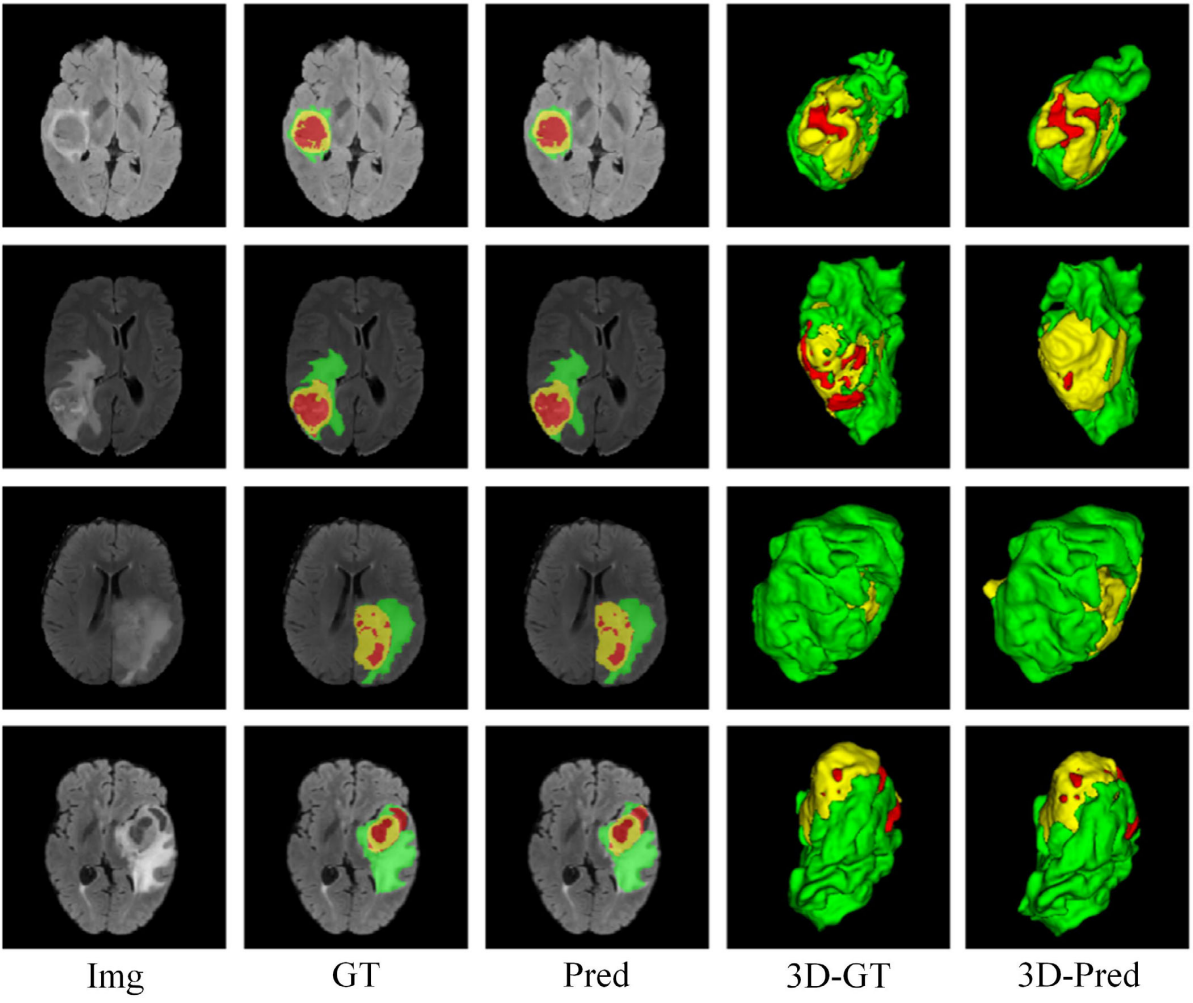
Segmentation examples from the training set.

To evaluate the segmentation results more effectively, we utilize visualization to display the tumor region segmentation with distinct colors for each subregion: green for edema, yellow for enhancing tumors, and red for necrotic regions. The segmentation outcomes are shown in Figure 4, with Img as the input image, GT as the GT map created through expert manual segmentation, Pred as the test result, 3D‐GT as the three‐dimensional representation of the GT map, and 3D‐Pred as the three‐dimensional display of the test outcome. For a more explicit demonstration of the model's segmentation capabilities, we randomly select some cases from the training set. The results, depicted in 2D and 3D, reveal that the model's predicted segmentation is highly similar to the GT, especially in the WT region, demonstrating precision and accuracy. With advanced applications, the model can effectively segment the subregions of brain tumor. However, some edge details may be blurred due to the lack of noticeable features.

#### 5.1.2 OS prediction results

Our study focuses on segmenting MRI scan images of patients with GBM. The goal is to accurately distinguish the tumors and subregions from normal brain tissue and use the segmentation results to predict the patient's OS time. To achieve this, a combination of three types of features is used: deep features obtained from a CNN, radiomic features extracted using the PyRadiomics toolbox, and clinical parameters such as age and tumor grade taken from the dataset. The OS time prediction results of this combination are demonstrated on the BraTS2020 dataset, as shown in Table 4.

**TABLE 4:** Results of OS time prediction on the BraTS2020 dataset.

Data set	MSE	MAE	RMSE
Training	129269.5032	251.5015	350.7228
Validation	139571.9641	254.6866	360.8906

We utilize evaluation indicators commonly used in regression algorithms, such as MSE, MAE, and RMSE, to assess the accuracy of the OS prediction model. These indicators measure the deviation between the predicted and actual values. A smaller value of these indicators indicates a closer match between the predicted and actual values, demonstrating the improved accuracy of the model. Table 4 shows that the prediction performance, as indicated by the evaluation indices, is high for both expert‐segmented training data and validation data segmented by the model. However, it is common for the prediction performance to be better for the training data than the validation data, as the tumor region in the expert‐segmented mask image is more accurately defined and the accuracy of the segmentation has a significant impact on the prediction outcome. By using the mask image as the research object, the predictions are more accurate with fewer errors.

The model's ability to perform well on various datasets is demonstrated by testing it on the BraTS2019 and BraTS2018 datasets, as shown in Table 5. The results show that the proposed model exhibits high prediction accuracy of the OS time on both datasets. The superiority of the training data over the validation data in terms of prediction accuracy is again highlighted. The importance of accurate tumor region segmentation is reinforced.

**TABLE 5:** Results of OS time prediction on the BraTS2019 and BraTS2018 datasets.

Dataset		MSE	MAE	RMSE
BraTS2019	Training	119924.3056	237.1829	337.6878
	Validation	121095.8173	237.6304	337.3586
BraTS2018	Training	114094.1324	228.7621	321.6653
	Validation	132036.0251	241.9858	350.1011

## 6 DISCUSSION

The training and testing of the proposed model produced pleasing segmentation results. After evaluating 125 examples, the target regions are determined, and the overall outcomes are in line with expectations. To offer a fairer comparison, we contrast our strategy with other methods. Experimental results on the BraTS2015 show that the average Dice for the top 15 teams employing this strategy is 0.8577. The majority of these teams utilize combined models, which improve image processing performance by merging image processing components into a single model. For instance, Sun et al.^17^ combined modules like Cascaded Anisotropic in three individual CNNs. Although there is still a gap with some of the current methods, they still obtained subpar results.

**FIGURE 5:**
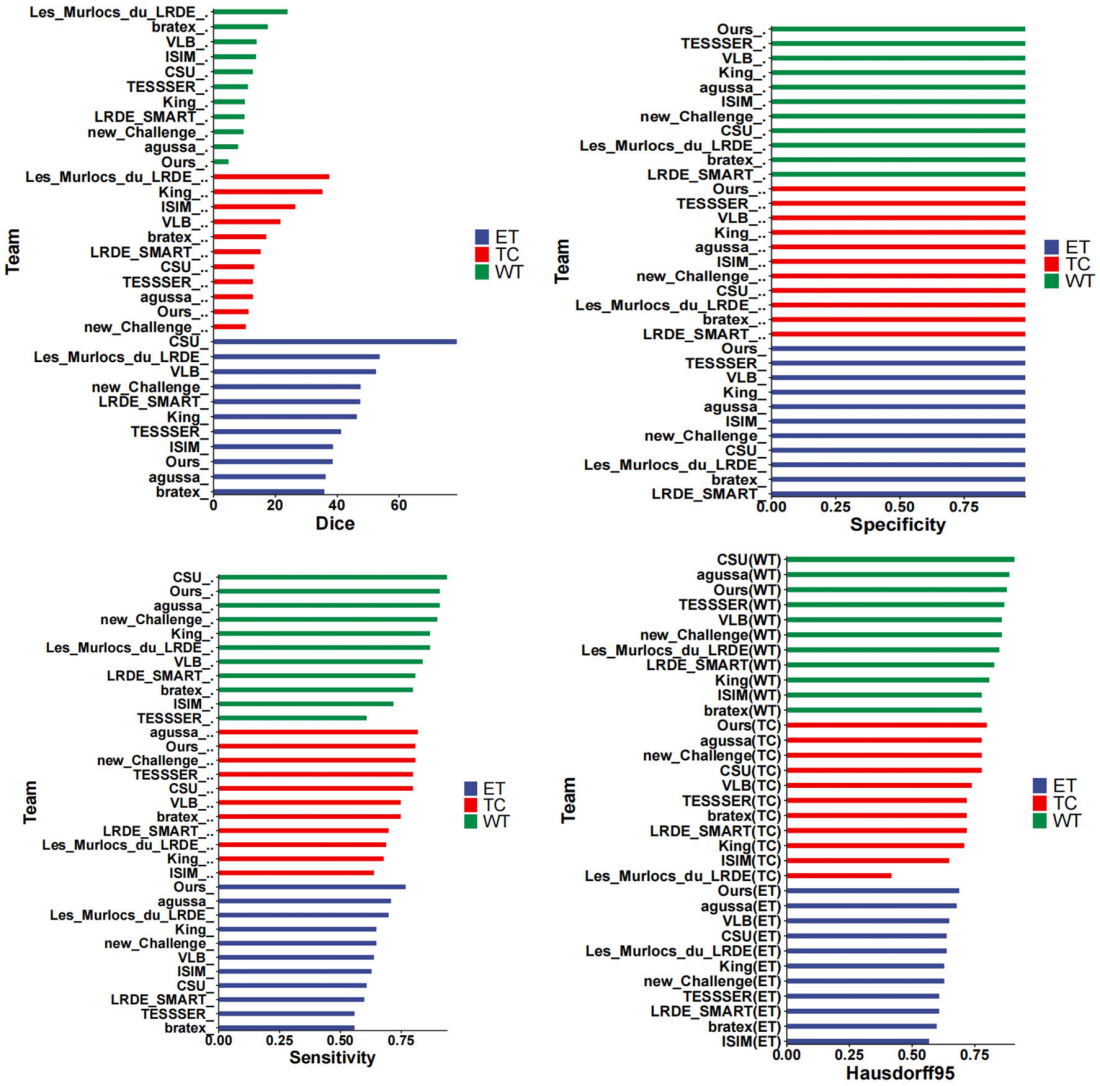
Comparison of segmentation results on the BraTS2020 dataset with those produced by other teams.

Zhao et al.^29^ found that joint training of two‐scale CNNs leads to a noticeable improvement in tumor classification accuracy compared to single‐path CNNs. Their results reveal that the joint training of two‐scale CNNs provides a substantial enhancement in the accuracy of tumor classification compared to single‐path CNNs. Most of the combination techniques in current research are based on the original technology or improved versions of it. Our segmentation results on the BraTS2020 dataset are compared with those of other teams^30^, ^31^, ^32^ in Figure 5, showing that our approach has higher accuracy and progression than these methods.

The accurate segmentation of brain tumors in MRI scan data plays a crucial role in determining tumor diagnosis and formulating prognostic plans. Although manual segmentation is still commonly used in clinical practice to gather information such as tumor size and location, it is prone to subjectivity and can be time‐consuming. As a result, the search for automatic segmentation techniques that increase efficiency and accuracy continues. Most current techniques are limited to 2D segmentation, while brain MRI images are three‐dimensional. It is recommended to use a 3D model for processing these images, which can result in more precise segmentation. Many experts and academics have conducted research on this topic and made significant contributions, including the development of new models such as 3D‐CNN,^33^ Cascaded Deep CNNs, and others.

The experimental results of the OS time obtained in this study are objectively evaluated by comparing them with the prediction results from other methods found in the relevant literature. The comparison data is presented in Table 6. Kim et al.^34^ employed radiomic features to make survival duration predictions using a random forest regression model. The features were first screened using a random forest method to avoid overfitting. Amian et al.^35^ used a random forest model to predict survival time and extracted spatial features from the entire tumor and its subregions. Kofler et al.^36^ made predictions solely based on the clinical feature of age and three orthogonal polynomial and orthogonal regression models. The XGBoost approach was used by Islam et al.^37^ to predict survival based on tumor geometry and a combination of location of radiomics features and clinical features. Soltaninejad et al.^38^ made predictions using a random forest model and features such as mean tumor intensity and the proportion of tumor volume to brain tissue. Agravat et al.^39^ used three features derived from age, volume, and shape to predict patient survival with a random forest regression model. Patel^40^ utilized a PCA to reduce the number of dimensions and a segmentation network to extract 2048 deep image features, then used a Cox hazard proportional model for survival prediction. Ali et al.^41^ predicted survival using a random forest recursive method to remove multiple radiomics features from MRI images and a grid search and random forest prediction model.

**TABLE 6:** Comparison of OS time prediction results with various methods.

Methods	MSE
Kim S. et al.^34^	121778.60
Islam M. et al.^37^	127478.65
Soltaninejad M. et al.^38^	109564.00
Agravat R.R. et al.^39^	116083.48
Patel J. et al.^40^	152467.00
Ours	121095.81

The results presented in Table 6 clearly show that the algorithm proposed in our study has several distinct advantages, as evidenced by the close alignment between the predicted results and actual values. This is a clear indication of the effectiveness of our proposed algorithm and provides a solid foundation for the clinical diagnosis and treatment of GBM and the advancement of personalized medicine. Despite the strengths of our proposed algorithm, it is important to note that there are some studies that have achieved even better prediction performance. This highlights the significance of continued optimization of the model in future development. By doing so, we can enhance the versatility of the model, minimize the prediction error, and achieve even greater accuracy in segmentation. Through these efforts, we can ensure that our proposed algorithm remains at the forefront of GBM diagnosis and treatment, providing the best possible outcomes for patients.

The analysis of the above results indicates that radiomic features have a strong tendency to provide stable and reliable outcomes, as well as having some interpretive advantages. Researchers and specialists have conducted an extensive experiment with two new image features that were derived from spatial and brain segmentation maps and discovered their usefulness in the field.^42^ If these two features are considered for inclusion in future studies, it can further enhance the prediction performance for survival. By combining the knowledge and expertise of clinical professionals, the accuracy of classifying radiomic features can be increased, thereby facilitating the identification of more precise feature selection techniques. The result of these efforts is a process that is both interpretable and easily applicable in a clinical setting, making it highly valuable for both researchers and practitioners alike.

## 7 CONCLUSION

In this study, we propose a new, automated framework for segmenting multimodal MRI scans and predicting the OS time of patients with GBM. The framework consists of two key components: a modified 3D‐UNet model that segments three subregions of GBM in multimodal MRI scans, and an SVR model that predicts patient OS time based on the extracted radiomic and clinical features. To use the framework, the first step is to segment the GBM subregions in the multimodal MRI scans with the modified 3D‐UNet model. Next, the radiomic features of the GBM tumor are extracted and combined with relevant clinical features, and the combined features are fed into the SVR model to make a prediction of the patient's OS time. The framework is tested and validated using datasets from the Brain Tumor Segmentation (BraTS) challenge. The results of the OS time prediction on the BraTS2020 dataset show an MSE of 139571.9641, an MAE of 254.6866, and a mean root square error of 360.8906, indicating that the proposed framework can predict patient survival from GBM multimodal MRI scans with a high degree of accuracy and precision. Overall, the proposed framework provides a novel approach to the segmentation of GBM multimodal MRI scans and the prediction of patient OS time, with the potential to have significant clinical implications for the early diagnosis of brain tumors.

## AUTHORS CONTRIBUTIONS

J. Zhu, J. Ye, G. Yang and X. Lai conceived and designed the study. J. Zhu, L. Dong, X. Ma, N. Tang, G. Yang, and X. Lai contributed to the literature search. J. Zhu, P. Xu, W. Jin, R. Li, G. Yang and X. Lai contributed to data analysis and data curation. J. Zhu, J. Ye, X. Ma, G. Yang, and X. Lai contributed to data visualization. J. Zhu and J. Ye contributed to software implementation. J. Zhu, X. Ma, N. Tang, P. Xu, W. Jin, G. Yang, and X. Lai contributed to the tables and figures. J. Zhu, L. Dong, X. Ma, R. Li, and X. Lai contributed to the writing of the report. J. Zhu, J. Ye, L. Dong, X. Ma, G. Yang and X. Lai contribute to the review and editing. All the authors have read and approved the publication of this work.

## CONFLICT OF INTEREST STATEMENT

The authors declare no conflicts of interest.

## Data Availability

The datasets analyzed during this current study are available in the BraTS2018, BraTS2019 and BraTS2020. https://www.med.upenn.edu/sbia/brats2018/data.html; https://www.med.upenn.edu/sbia/brats2019/data.html; https://www.med.upenn.edu/sbia/brats2020/data.html.
